# A Breath of Fresh Air: Perspectives on Inhaled Nutrients and Bacteria to Improve Human Health

**DOI:** 10.1016/j.advnut.2024.100333

**Published:** 2024-10-30

**Authors:** Flávia Fayet-Moore, Stephen R Robinson

**Affiliations:** 1 FOODiQ Global, Sydney, NSW, Australia; 2 School of Environmental and Life Sciences, the University of Newcastle, Ourimbah, NSW, Australia; 3 Discipline of Psychology, School of Health & Biomedical Sciences, Royal Melbourne Institute of Technology, Bundoora, VIC, Australia; 4 Institute for Breathing and Sleep (IBAS), Austin Health, Heidelberg, VIC, Australia

**Keywords:** aeromicrobe, aeronutrient, gastrointestinal tract, lung, microbiome, nature, nutrition

## Abstract

We propose that the human respiratory system and olfactory pathways sequester airborne nutrients (vitamins, fatty acids, and trace minerals) that are beneficial for health, which we term “*aeronutrients.*” In addition, airborne bacteria, termed “*aeromicrobes*,” have the potential for positive health effects by improving species diversity in the microbiotas of the respiratory and gastrointestinal tracts. These concepts have implications for people living in urban areas or those who have limited access to nature, such as astronauts exposed for long periods to highly filtered air that may be depleted of aeronutrients and aeromicrobes. The possibility that fresh air contributes to human nutrition and health may stimulate a re-evaluation of guidelines pertaining to nutrition and access to natural environments, and will open new avenues of scientific enquiry.

**Statement of Significance**

We propose that the human respiratory system sequesters airborne nutrients that are beneficial for health, and that airborne bacteria improve species diversity in the airways and gut. These novel concepts, which are strongly supported by the available data, have the potential to stimulate a re-evaluation of guidelines pertaining to nutrition and access to natural environments, and open new avenues of scientific enquiry.

## Introduction

Throughout an average lifetime, we inhale ∼438 million liters of air [1], a volume equivalent to 175 Olympic-sized swimming pools. In addition to oxygen, nitrogen, and other gases, each breath contains small amounts of particulate matter, trace elements, as well as soluble and volatile molecules. As some of these have bioactive properties, is it possible that an alternate source of nutrients has been literally right under our noses all along and we failed to notice it?

For centuries, it has been considered that the nutrients needed for healthy growth, functioning, and repair are obtained exclusively from our diet. This concept was challenged in 2019 by Trayhurn [2] who argued that oxygen should be considered a critical nutrient. He noted that oxygen had been ignored by nutritionists because it enters the body via the respiratory system rather than the gastrointestinal tract (GIT). Trayhurn asserted that “*the route of entry should not be the critical factor in defining whether a substance is, or is not, a nutrient.*” If we accept this definition, then we ought to consider whether air contains anything other than oxygen that could serve as a nutrient or is beneficial for health. The present article advances this notion, and from here on, we refer to these airborne nutrients as “aeronutrients.” We envisage that some aeronutrients are naturally present in fresh air, whereas others are added by human activities, such as cooking or nebulization. In both cases, they need to be present at concentrations sufficient to provide measurable physiological, cognitive, and/or health benefits when inhaled. In a related concept, we note that when inhaled, airborne bacteria can replenish the populations of commensal bacteria living in the airways. Some of these bacteria enter the GIT where they also replenish the gut microbiome and aid digestion.

**FIGURE 1:**
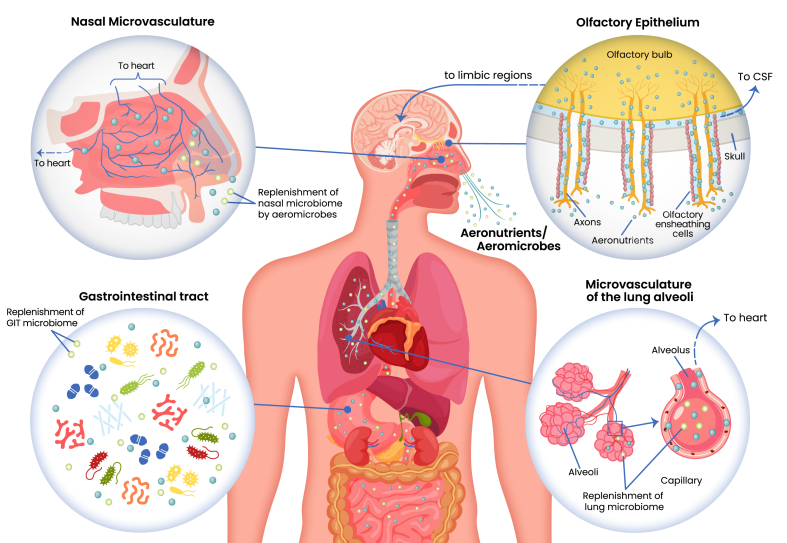
Routes of entry into the human body by aeronutrients and aeromicrobes. Aeronutrients have 3 primary routes of entry into the body: *1*) Inhaled micronutrients are absorbed through the microvessels lining the nasal cavity that drain into several veins that take blood directly to the heart. *2*) The olfactory epithelium at the apex of the nasal cavity enables aeronutrients to be transported along olfactory axons into the olfactory bulb and thence to limbic regions of the brain, bypassing the blood–brain barrier. In addition, aeronutrients can diffuse through the fluid-filled spaces surrounding the axons that lead into the cerebrospinal fluid (CSF) of the brain. *3*) The lung alveoli are a site of entry for aeronutrients. Capillaries lining the alveoli have a massive absorptive surface that transports aeronutrients directly to the heart. Aeromicrobes have 2 primary routes of entry into the body: *1*) Aeromicrobes seed and replenish populations of commensal bacteria in the nasal cavities and respiratory pathways. *2*) Aeromicrobes in the oropharynx can enter the gastrointestinal tract by ingestion or by mucociliary clearance to seed the gut microbiome. Dark green circles = aeronutrients; Light green circles = aeromicrobes. CSF, cerebrospinal fluid.

The evidence that inhalation can result in the uptake of bioactive molecules into the circulatory system and brain is extensive. The various routes of uptake are the nasal microvasculature, the olfactory epithelium, the microvasculature of the lung alveoli, and the GIT via the oropharynx (Table 1 and Figure 1), discussed in detail in the following sections, including how they can relate to the uptake of aeronutrients.

**TABLE 1:** The principal routes by which aeronutrients and aeromicrobes may enter the human body.

Tissue	Type	Route of entry	Target
Olfactory epithelium	Aeronutrients	Via fluid in the extracellular space between the olfactory nerves, and then into the cerebrospinal fluid	Entire brain
Olfactory epithelium	Aeronutrients	Along the olfactory nerves to the olfactory bulb, then along the olfactory tract to associated forebrain structures	Limbic areas of brain
Olfactory epithelium	Aeronutrients	Along the trigeminal and facial nerves, to their ganglia, then to associated nuclei	Cranial nerve nuclei in brainstem
Nasal microvasculature	Aeronutrients	Across the walls of the capillary bed into the venous circulation, bypassing the hepatic-portal circulation	All organs
Nasal cavity	Aeromicrobes	Microbial seeding and replenishment of the nasal microbiome	Nasal cavity
Lung alveoli	Aeronutrients	Across the walls of the capillary bed into the venous circulation, bypassing the hepatic-portal circulation	All organs
Airways and lung alveoli	Aeromicrobes	Seeding and replenishment of the airway microbiome during breathing	Airways
Oropharynx and nasal cavity	Aeromicrobes	Seeding of the GIT microbiome during ingestion of food or mucociliary clearance from the oropharynx	Gastrointestinal tract

### Nasal microvasculature

Although the primary purpose of the nose is to mediate olfaction, an equally important function is to filter, heat, and humidify inhaled air so it does not damage the delicate lung epithelium. The nasal cavity consists of 3 pairs of turbinates, stacked behind the septum, that expand the combined internal surface area of the 2 nasal cavities to ∼150 cm^2^ [3]. Much of this surface is lined by a dense capillary plexus that is separated from the air by a single layer of epithelium (Figure 1). The extensive blood flow through this plexus warms and humidifies air passing over it [4]. However, this plexus is vulnerable to infiltration by airborne lipophilic molecules that can dissolve into the lipid walls of the capillaries (transcellular route), and infiltration by aqueous molecules smaller than 1000 Daltons that can diffuse through the spaces between adjacent endothelial cells (paracellular route). Below 1000 Daltons, absorption is inversely related to molecular size, and positively correlated to concentration, hydrophilicity, and lipophilicity [5,6]. In addition, nasal capillary endothelial cells express a variety of membrane-bound transporters that bring amino acids, trace elements, amines, and cations from the nasal cavity into the blood [5,7].

The nasal capillaries drain into veins connected to the systemic venous circulation that carries blood directly to the heart, bypassing the hepatic-portal circulation (Figure 1). Known as the “first pass effect,” intranasal administration of therapeutic drugs can lead to more rapid and higher systemic concentrations than when those same drugs are delivered orally. The nasal microvasculature has become an important target for the systemic delivery of a wide range of therapeutic agents, including analgesics, antiemetics, insulin, sedatives, antiseizure medications, and migraine treatments [6,8]. The effectiveness of this route is demonstrated by nasal sprays of naloxone, an opioid receptor antagonist, which has become a first line of emergency intervention in life-threatening cases of opioid intoxication, such as heroin overdose [9]. Several recreational drugs, including cocaine hydrochloride and methamphetamine, are delivered by “snorting” the powdered drug into the nasal cavity where it is absorbed into the nasal microvasculature [10]. The uptake of cocaine is extremely rapid, with physiological and cognitive effects beginning 3–5 s after administration [11]. Although these drugs are not aeronutrients, they demonstrate the effectiveness of the nasal microvasculature for the quick and direct transfer of airborne molecules into the bloodstream.

### Olfactory epithelium

The apex of the nasal cavity contains the olfactory epithelium, consisting of around 10 million olfactory receptor neurons and a dense plexus of their cilia, which subserve the sense of smell. Olfactory receptor neurons send their axons through holes in the skull to terminate directly on the olfactory bulb, which lies within the cranium (Figure 1) [12]. The olfactory receptor neurons are the only part of the brain that interfaces directly with the external environment. They are protected from desiccation by a layer of mucus, and their cilia are studded with receptors, each with a binding affinity for specific odorant molecules [12]. The cilia also express a wide range of transporters for potential aeronutrients including organic cations and anions, zinc, divalent metals, dopamine, and amino acids [5,7].

In the brain, gaps between adjacent vascular endothelial cells are uniquely sealed with rows of tight junctions, which prevent most solutes in the blood from diffusing into its neuropil. This blood–brain barrier excludes harmful toxins and pathogens, and also prevents blood-borne therapeutic agents from entering the brain [12]. The axons of the olfactory receptor neurons circumvent the blood–brain barrier. If solutions of drugs are applied to the surface of the olfactory epithelium, they diffuse along the olfactory axons into the olfactory bulb and then deeper into the brain or cerebrospinal fluid. This “nose-to-brain” route has been a topic of intense research, and is an efficient pathway for aeronutrients to reach the brain.

Drugs and other exogenous molecules have been shown to move along the nose-to-brain route via intracellular and extracellular pathways (Figure 1) [13]. The intracellular pathway involves internalization of the molecule by olfactory receptor neurons either through the action of membrane-bound transporters, endocytosis, or in the case of lipophilic molecules, by dissolving into the cell membrane. The molecule then moves along the axon and is released into the olfactory bulb. The extracellular route involves dissolution of the molecule in the fluid surrounding the axons of the olfactory receptor neurons, and its subsequent diffusion into the cerebrospinal fluid surrounding the olfactory bulb, and thence to other brain regions. In addition to the olfactory nerve, the walls of the nasal cavity are innervated by somatosensory branches of the trigeminal nerve and motor branches of the facial nerve. However, these nerves are less effective for the nose-to-brain transport of drugs [14], probably because transport is retarded by intervening ganglia and a longer distance. This limitation is likely to apply to aeronutrients as well.

Data from mouse models indicate that intranasally administered drugs reach the olfactory bulb within 5 min and attain a peak concentration 10 min after administration. More distant locations, such as the hypothalamus, hippocampus, striatum, and midbrain, reach peak concentrations 30–60 min after administration [13]. Comparable observations have been obtained in human brain imaging studies [15]. Further, in mouse models of Alzheimer’s disease and in clinical patients with mild cognitive impairment or early-stage Alzheimer’s disease, intranasal doses of insulin improve attention, memory, and cognitive function [13,16]. Therapies with clinical approval for administration through the nose-to-brain route include insulin for posttraumatic stress disorder and mood disorders, glutathione for Parkinson’s disease, and oxytocin for autism spectrum disorder and schizophrenia [15].

### Olfactory nutrient absorption

The dendrites of olfactory sensory neurons express a variety of metal transporters, and there is evidence that the essential nutrients manganese and zinc can enter the brain via the olfactory epithelium [7,17]. Manganese is of particular interest, because it is the 12th-most abundant element in the Earth’s crust and is naturally present in the atmosphere at an approximate concentration of 15 ng/m^3^ [18]. Manganese is a cofactor of glutamine synthetase, the enzyme in astrocytes that detoxifies both glutamate and ammonia [19]. Brain imaging and tracer studies show that blood-borne manganese is excluded by the blood–brain barrier, and instead it enters the brain via blood vessels supplying the choroid plexus [20,21], or via the nose-to-brain route [7,22]. Although an essential micronutrient, high concentrations of manganese are neurotoxic, and the nose-to-brain route is associated with the accumulation of neurotoxic levels of manganese in individuals exposed to excessive airborne concentrations, such as welders [19,23]. This finding demonstrates that airborne manganese, an aeronutrient, is transported into the brain from the nasal epithelium and raises the possibility that under normal atmospheric concentrations this route provides the brain with a significant amount of this essential nutrient, potentially at much lower concentrations than required from the diet.

Another essential aeronutrient of interest is iodine, a key constituent of the thyroid hormones thyroxine and triiodothyronine, which play vital roles in human brain development and metabolism. Most soils are deficient in iodine, and consequently, with the exception of seaweed and fish, diet is an inadequate source of iodine. In 1993, the WHO reported that the majority of the global population was iodine deficient; this dire situation was partly rectified by the worldwide introduction of iodized salt [24], though it remains an issue in many countries. In 1964, Vough et al. [25] estimated that levels of iodine gas in the atmosphere could potentially provide a daily iodine intake of 11 μg/d, or 7.3% of the recommended dietary intake for adults of 150 μg/d [26]. They then examined laundry workers who were routinely exposed to high atmospheric concentrations of iodine [25]. They found that the levels of iodine in the serum and urine of these workers were substantially higher than those in a control group of workers and suggested that atmospheric iodine can be absorbed through respiration, although they could not exclude other routes of uptake.

Then, in 2011, Smyth et al. [27] were interested in the contribution of iodine gas taken through respiration in supplying a proportion of daily iodine requirements. They hypothesized that inhaling iodine gas released from seaweed would contribute to daily iodine intake. They first measured gaseous iodine release over seaweed beds by gas chromatography, then measured iodine intake via urinary iodine excretion. The urinary concentrations of iodine in Irish female schoolchildren under 15 y, living in 3 locations: coastal with abundant seaweed present in the environment (*N* = 28), coastal with little seaweed present (*N* = 93), and inland areas of Ireland were compared (*N* = 46) [27]. All groups had a low iodine content in their diets, including an absence of iodized salt. Smyth et al. [27] found that the concentrations of atmospheric iodine in seaweed-rich locations were ∼11-fold higher than background levels. Furthermore, urinary concentrations of iodine were 2.7-fold higher in the children living in seaweed-rich areas when compared with the other 2 locations, and the proportion of children with probable iodine deficiency was 8.7%, 14.5%, and 37.6% in the seaweed-rich, seaweed-poor, and rural groups, respectively. The authors suggested that residents of seaweed-rich areas may obtain a significant proportion of their daily iodine requirement by inhalation from seaweed, rather than proximity to the ocean, although further research with greater control of dietary intake was required to confirm this. Thus, airborne iodine appears to be another example of an aeronutrient that can supplement dietary sources.

### Lung alveoli

To facilitate the transfer of oxygen into, and carbon dioxide out of, the bloodstream, the walls of alveoli in the lungs are densely packed with capillaries that are separated from the air by a layer of fluid less than a micron thick. The combined area of the alveolar walls is around 100 m^2^ [28,29], providing a huge surface for gas exchange. In addition to oxygen, other airborne molecules and particles are able to cross into the bloodstream (Figure 1), largely through the processes of passive diffusion and transcytosis [28,29]. Small, mildly hydrophobic molecules such as nicotine, cannabinoids and opioids are absorbed into the systemic circulation within seconds. Larger and highly hydrophobic molecules such as insulin and transretinoic acid (an active metabolite of vitamin A) are absorbed over intervals of minutes to hours [28,30], suggesting that this vitamin may also be an aeronutrient, as it occurs naturally at the air–water interface of rivers, lakes and the ocean [31].

Throughout human history, people have inhaled drugs, primarily in the form of smoke, attesting to the effectiveness of the lung epithelium for transporting bioactive molecules from the air into the bloodstream. Although inhalational anesthesia was introduced in the mid-nineteenth century, it is only in the past 2 decades that Western medical science has begun to explore the suitability of this pathway for the administration of therapeutic drugs into the systemic circulation [32,33].

Bulk dietary requirements of macronutrients, such as carbohydrates, amino acids, and water are obtained primarily, if not exclusively, from the GIT, whereas the lungs are more suited for the uptake of larger molecules. In comparison to the GIT, the lungs contain fewer proteases, have a less acidic pH, and have fewer efflux transporters for xenobiotics, which means that inhaled peptides and proteins are more likely to reach the bloodstream intact than if they had been delivered orally [28]. Furthermore, the GIT is impermeable to molecules larger than 600 Daltons, whereas the lung epithelium has uptake mechanisms for nanoparticles [29,34] and for molecules as large as 160,000 Daltons [28], enabling absorption of a wide range of potential aeronutrients. Finally, like the nasal microvasculature, blood leaving the alveoli goes directly to the heart, bypassing the hepatic-portal circulation. This means that drugs absorbed through the alveolar epithelium, unlike those taken up from the GIT, are not sequestered and detoxified by the liver, requiring a far lower dose of the drug (or nutrient) to achieve the same potency and blood concentration.

Clinical research conducted 70 y ago [35] first demonstrated the effectiveness of aerosolized vitamin B_12_ for the treatment of pernicious anemia. In a clinical study of 24 patients, Shinton and Singh [36] showed that aerosolized vitamin B_12_ is 54-fold more efficient than oral supplementation and almost as efficient as intramuscular injections. These findings have been confirmed by subsequent research. For example, a study of 10 children with vitamin B_12_ deficiency [37] reported that a nasal spray of vitamin B_12_ rapidly normalized serum levels, whereas 103 vitamin B_12_-deficient adults treated with nebulized vitamin B_12_ all attained normal serum levels within 2 wk and remained stable thereafter [38]. Recently, a study of 60 vitamin B_12_ deficient elders (>65 y old) reported a rapid improvement in serum levels following regular treatment with an intranasal spray of vitamin B_12_ [39].

A pharmacokinetic study of 10 elderly patients [39] demonstrated that 1500 μg of inhaled vitamin B_12_ is rapidly incorporated into the bloodstream, rising from a baseline of 130 pmol/L to reach a peak of 3500 pmol/L in just 28 min. They reported that the probable mechanism of uptake is via aqueous channels in the nasal epithelium, although the presence in nasal secretions of haptocorrin, a transporter of vitamin B_12_, implies that a transporter-mediated pathway may also be involved. Because inhaled vitamin B_12_ aerosols are demonstrably safe and effective, further research is needed to determine whether the nebulization of other micronutrients, such as vitamin D, can assist in addressing global deficiencies.

Like nebulized vitamins, peptides and protein drugs have considerable potential for the treatment of disease because of their high potency and specificity, and lower toxicity compared with conventional drugs [34]. However, such drugs must be injected into the body because they are degraded in the GIT if taken orally. The relative ease with which inhaled peptides and proteins can enter the bloodstream has stimulated research into the inhalational delivery of such drugs. Researchers have found that the mucosal barriers to protein transport in the lung can be circumvented if the drugs are encapsulated within nanoparticles of fatty acids, such as linoleic or oleic acid, resulting in substantially enhanced absorption [21]. These particles, typically composed of a linoleic acid or oleic acid shell and an aqueous core, can remain in the air for several days after being generated [40,41], and have the potential to become aeronutrients that contribute to the intake of essential fatty acids. There have been no investigations, to our knowledge, of the potential for aerolized proteins to contribute to nutrition, despite the fact that the cooking of food releases fatty acid nanoparticles into the air. Until now, research has focused on the potential of inhaled nanoparticles to adversely affect human health [42]. This focus overlooks the possibility that lipid nanoparticles released during the cooking of food may contain essential macronutrients and bioactive peptides that can be absorbed through the lungs into the bloodstream, as a supplemental source of nutrition.

### The aerobiome

In normal parlance, “fresh air” is used to differentiate the air outdoors that is perceived to be clean and free from indoor contaminants from the stuffier, stale air often found inside occupied living spaces. Fresh air does not have a scientific or universal definition. It can be defined as air in urban environments that meets standards for air quality, with the levels of dust, pollens, and pollutants being below specified thresholds or as air that is found in natural environments like parks, forests, or near bodies of water. For the purposes of the present review, fresh air is typified by that found in pristine natural or rural environments and is minimally contaminated by harmful microbes or pollutants from anthropogenic sources. Although fresh air contains microbes, particles, aeronutrients, and volatile chemicals from nonhuman sources, it is not regarded as fresh if contaminated by smoke, volcanic gases, or malodors. Conversely, the highly filtered, recycled air found in submarines and space stations is not fresh, despite its cleanliness and odorlessness, because it can lack aeronutrients and aeromicrobes.

The health-giving properties of fresh air have been appreciated for millennia, with Roman writers promoting this concept from the second century BC onward [43]. For centuries, Scandinavian cultures have adhered to the principle of “*Friluftsliv*,” which encourages people to spend time outdoors, even in winter, as it contributes to better health [44]. Research has shown the benefit of immersive nature experiences on mental health (forest bathing and nature therapy) [45,46] and the immune system [47], yet the mechanistic basis of these benefits remains unclear. “Natural environment,” “natural settings,” or “nature” are used interchangeably to describe outdoors locations that are relatively unaffected by human activity, such as forests, alpine areas, or undeveloped coastline, but they can also refer to vegetated greenspaces or parks in urban centers. In either case, individuals exposed to nature and urban green spaces for more hours per week are healthier [45,48], access to green space improves mental health in children [49], and the immersion of children in nature improves the composition of their microbiota and their behavior [50]. Although the health benefits of fresh air in natural spaces are partly because of a reduction in airborne industrial pollutants, additional benefits may come from the greater diversity and abundance of aeronutrients and aeromicrobes in natural settings. While some aeromicrobes are pathogenic, the majority are harmless and some can be beneficial for health.

Bacteria in soil or water, on vegetation or exhaled by animals, are lifted into the air by wind, spray, and evaporation. The aerobiome of a place reflects the microbiome of the locality, and the species composition of an aerobiome varies substantially between habitats and in its vertical stratification [47]. A comparison of 9 global sites reported that bacterial concentrations range from 9.2 × 10^1^ to 1.3 × 10^8^ cells per cubic meter of air [51]. These values were mostly correlated with the surrounding landscape, and were highest in natural settings and lowest in urban sites, meaning that people living in rural locations inhale several million bacteria every minute whereas city dwellers may inhale just a few hundred.

### Aeromicrobes seed microbiotas

Most surfaces and cavities of the human body contain colonies of bacteria, fungi, and viruses. These microbes are collectively termed the microbiota, and along with their metabolites and microecological niche that they occupy, are collectively termed the microbiome [52]. Because relatively little is known about commensal viruses and fungi, only commensal bacteria will be discussed here.

As the nasal and oral cavities are in direct contact with the external environment, they harbor high densities and diversity of bacterial species [53]. Neonates acquire basic oral and nasal microbiotas from their mothers [54], and during infancy species diversity and numeric density increase because of the colonization by bacteria from the local environment. By 2–3 y of age, these microbiotas have adapted to their host, stabilized, and become fairly resistant to colonization by new species, although shifts in microbiota composition continue throughout life and are affected by environmental factors, such as seasonal changes and air pollution [55,56].

Bacteria from the nasal and oral cavities and upper respiratory tract are source populations for the lung microbiota [53]. Consequently, there are no bacterial species present in the lungs that are not also present in the upper airways [57]. Bacterial diversity and concentration progressively decrease from the upper to lower airways because of the limitations of dispersal and clearance by macrophages [57]. Breathing and coughing expel bacteria from the nasal and oral cavities, as well as from the lungs.

Aeromicrobes are transmitted between people indoors [58] and those living in proximity can exchange microbes, leading to a convergence of microbiota composition. The gut microbiotas of spouses are more like each other than those of related or unrelated individuals, suggesting that the exchange of gut microbes occurs through close contact [59]. Studies on the International Space Station, where there is no fresh air, show that microbial transfer occurs between astronauts, and that the composition of their microbiota is more similar during space flight than postflight [60]. There is a reduction in nonpathogenic bacteria in the nasal cavity and an increase in pathogenic bacteria, such as *Staphylococcus;* this shift in composition persists for 2 mo after the astronauts have returned to Earth [61].

The microbiota of the GIT has been extensively studied, and its contributions to nutrition, defense against pathogens and immune modulation are well established [62]. Commensal bacteria release factors that interact with immune cells at the intestinal barrier to reinforce barrier immunity and protect the microbiome from opportunistic pathogens [63]. These bacteria are also responsible for the *de novo* synthesis of several essential micronutrients (e.g. B group vitamins, vitamin K, short chain amino acids), they increase the bioavailability of several other micronutrients (e.g. vitamins C, D, and E, calcium, iron, and phosphate), and are a major determinant of human micronutrient status [64].

The microbiota composition of the GIT varies substantially along its length, because of microenvironmental differences such as pH, oxygen tension, and the type of nutrients available [65]. In addition, microbial diversity and abundance in the GIT is higher in individuals who are regularly exposed to natural environments, animals, and other people [56]. There is a substantial overlap between the phyla of bacteria found in air and those in the respiratory tract, the oropharynx, and the GIT [65,66]. Like the microbiome of the respiratory tract, *Bacteroidetes* along with *Firmicutes* comprise over 98% of bacteria in humans GIT [67]. Aeromicrobes strongly influence the composition of the nasal and oral microbiotas and in turn, these regions seed microbiotas in the lungs and GIT (Figure 1). Interestingly, bacterial cross-talk and transfer appears to occur in both directions, with multiple lines of evidence indicating that GIT bacteria can colonize the lungs and *vice versa*. Furthermore, lung disease is associated with dysbiosis in the GIT and conversely, bowel disorders result in dysbiosis of the respiratory tract (reviewed by Enaud et al. [62] and Li et al. [68]. These interrelationships make it likely that commensal bacteria of the airways produce essential nutrients for their host in a similar way to those in the GIT, although this speculation remains to be investigated.

Although exposure to natural environments is important during early childhood, when the microbiota populations are stabilizing, replenishment by benign bacteria is needed throughout life, particularly after exposure to factors that deplete these bacteria, such as antibiotics [69], airborne pollutants [70,71], ionizing radiation [72], and acute infections [73]. When compared with urban environments, rural environments are characterized by a greater diversity and numerical density of aeromicrobes, and this difference is reflected in a greater diversity of bacterial species in the nasal microbiota [74,75]. Regular exposure to natural environments contributes to greater species diversity in the respiratory pathways and confers better protection from pathogenic bacteria [76,77]. For instance, the nasal microbiotas of rural dwellers contain significantly higher proportions of nonpathogenic *Corynebacteriaceae* whereas urban dwellers have higher proportions of pathogenic *Staphylococcus aureus* [75]. Experimental studies have shown that continuous exposure to *Corynebacteriaceae* prevents the colonization of the nasal cavity by *Staphylococcus aureus* [78]. *Corynebacterium accolens* cultured from the nasal cavities of healthy human volunteers have potent antibacterial activity against *Staphylococcus aureus*, including drug-resistant strains of this pathogenic bacteria [76], and *Corynebacteriaceae* protect the nasal microbiome by scavenging iron required for the growth of *Staphylococcus aureus* [79].

A comparison of nasal microbiota in 43 healthy adults and 54 adults with chronic respiratory diseases found that the healthy group had higher levels of *Corynebacterium accolens,* as well as several other bacteria that are normally associated with plants and bees, suggesting that exposure to bacteria from natural environments may improve resistance to respiratory diseases [77]. Another study found that urban infants exposed to mild-moderate levels of air pollution had reduced levels of *Corynebacteriaceae* in their nasal microbiota, which in turn was associated with higher risks of asthma and respiratory disorders [80]. Furthermore, the prevalence and mortality rates of adult asthma in urban areas are consistently higher than in rural areas, despite rural areas having limited access to healthcare and an increased incidence of sensitizing agents such as pollens, dust mites, endotoxins, and biomass smoke [81]. Nonetheless, within urban environments, access to green spaces is associated with improved health outcomes. A 12-y prospective community study of 581,785 urban dwellers in China demonstrated a strong inverse correlation between the amount of exposure to green spaces and the risk of mortality from lung cancer [82].

When viewed together, the preceding observations demonstrate that people living in rural environments, as well as urban dwellers with regular access to green spaces, have better respiratory health, and this appears to be associated with the greater abundance and diversity of beneficial aeromicrobes in these environments [74,83].

### The good and the bad

Airborne bacteria such as *Staphylococcus aureus, Escherichia coli, Streptococcus pneumoniae*, and *Klebsiella pneumoniae* present serious threats to human health and collectively kill 3–4 million people annually [84]. To address these risks, research has focused on issues such as microbe survivability in the air, sanitation of public spaces, the development of coordinated pandemic responses, the prevention of bacteriological warfare, regulation of laboratory safety, and public health policies and guidelines that aim to reduce exposure to airborne microbes. Although these initiatives are essential, we should be cognizant that the infectiousness of airborne pathogens is facilitated by factors such as high density living, poor sanitation, international air travel, intensive animal husbandry, laboratory cell culture, and germ warfare. These factors are strongly associated with the presence of large urban centers, and the highest concentrations of airborne pathogenic bacteria are found in urban and suburban regions, especially near hospitals and wastewater treatment plants [85]. The requirement to contain the spread of airborne pathogens in urban settings has overshadowed the fact that in rural and remote settings, naturally occurring airborne bacteria promote health by supporting a more diverse microbiome.

In a similar vein, airborne nanoparticles and chemicals are widely regarded as being bad for health. The negative effects of anthropogenic airborne pollutants on human health are illustrated by the fact that airborne PM_2.5_ particulates originating from industrial or urban activities are linked to higher incidences of respiratory and systemic diseases and to increased rates of mortality [86,87]. However, as discussed earlier, some naturally occurring components of air, such as aeronutrients, are beneficial for health. A distinguishing feature of these aeronutrients is that they occur at relatively low concentrations, whereas air pollutants arising from human activities are not nutritive yet are transported into the body by the same mechanisms and metabolic pathways that likely evolved to acquire the more scarce aeronutrients from air.

The gills of fish are specialized for taking up dissolved minerals and lipids and bioconcentrating them to several orders of magnitude higher than are present in the water [88,89]. Absorption via the gills is a physiologically relevant route for the uptake of micronutrients, yet in fish that live in water polluted by industrial runoff, this mechanism can lead to the accumulation of toxic concentrations of heavy metals [88,89]. Similarly, in humans, chemicals, and nanoparticles released into the atmosphere by industry are absorbed via routes that likely evolved to facilitate the uptake of scarce aeronutrients. These pathways lack the specificity to distinguish between natural and artificial components in air, or between beneficial aeronutrients and aeromicrobes compared with pollutants and harmful aeromicrobes.

In some instances, industrial processes result in abnormally high airborne concentrations of aeronutrients, which in turn become a pollutant, and can lead to excessive uptake and toxicity. For example, a magnetic resonance imaging study of 2836 Italian children found that exposure to high levels of airborne copper was associated with significantly poorer reaction times and reduced connectivity between the caudate nucleus and frontal cortex [90]. The copper was not from fresh air, and originated from industrial activity, brake pads on motor vehicles and overhead wires on electric railway lines. Although the diet contains far higher concentrations of copper than the atmosphere, ingested copper is chelated by ceruloplasmin, enabling any excess to be excreted, whereas “*nonceruloplasmin-bound copper absorbed in the respiratory tract can more easily enter the brain and achieve higher tissue concentrations*” [90].

The foregoing discussion illustrates how aeronutrients and aeromicrobes can be both good and bad for human health. In environments with fresh air, unaffected by air filtration, industrial or urban activities, aeronutrients, and aeromicrobes uniformly promote health. However, toxic nanoparticles and chemicals released into the atmosphere by human activity can enter the brain, lungs, and bloodstream via the same mechanisms, summarized in Figure 1, that exist to promote health. Furthermore, urban environments are more favorable than rural environments to the transmission and proliferation of pathogenic aeromicrobes. At present, most public health policies fail to differentiate between pollutants and the naturally occurring components of air that contribute to human health. We recommend the adoption of a more nuanced approach to public health: one that aims to minimize exposure to anthropogenic pollutants while facilitating access to aeronutrients and beneficial aeromicrobes.

### Future directions

We propose that the act of breathing enables the acquisition of beneficial airborne nutrients, termed "aeronutrients" to distinguish them from the conventional “gastronutrients” absorbed through the GIT. In support of this concept, we reviewed clinical and experimental evidence showing that the capillary lining of the nasal cavity, the olfactory epithelium and the alveoli of the lungs are all specialized to acquire molecules including nutrients, from the air and transport them into the systemic circulation or directly into the brain. We consider it likely that aeronutrients supplement the dietary intake of some essential fatty acids, vitamins, and trace minerals. The available evidence indicates that airborne iodine, manganese, vitamin A, and vitamin B_12_ can contribute to nutritional status, whereas high concentrations of airborne manganese and copper from industrial activities can be toxic. As some of the studies supporting our thesis were conducted several decades ago, they will need to be confirmed with modern analytical techniques, while controlling for dietary intake. In addition, research is required to better understand the concentrations at which aeronutrients can be beneficial or harmful. As the field of aeronutrients evolves, we expect to uncover other examples of aeronutrients and beneficial components in air that have been undetected because of the prevailing focus on air pollutants.

The concept of aeronutrients introduces the possibility that fresh air contains bioactive molecules, such as peptides and proteins, that until now have not been considered to contribute to nutrition because the focus on nutrition has been on diet and the GIT. Support for this possibility comes from evidence that the lungs can transport intact peptides and proteins into the bloodstream, whereas the GIT cannot. This interesting possibility awaits further investigation, along with the bidirectional link between the GIT and lung and its effect on nutritional status.

We also propose that beneficial airborne bacteria, which we have termed aeromicrobes, seed the microbiotas of the airways and GIT, thereby enhancing species diversity, aiding digestion, and providing protection from pathogenic species. Research is needed to ascertain whether microbiotas in the nasal and oral cavities and lungs provide their host with essential nutrients, like those in the GIT. Further investigations are needed into the sharing of aeromicrobes between individuals, and the contributions that aeronutrients and aeromicrobes make to human health. A recent study demonstrated that when female mice were exposed to an aerobiome derived from soil, the microbiota of their GIT changed and they displayed less anxiety-like behavior [91]. Investigations such as this, employing animal models in environments with meticulously controlled air composition, alongside human studies in isolated, confined spaces utilizing closed-loop air systems such as submarines and space stations, may advance our understanding of the roles played by aeronutrients and aeromicrobes in human health.

Because airborne bacteria and other components of the aerobiome, such as pollen and phytoncides, can have beneficial effects for health, some researchers have emphasized the need to shift societal focus away from the adverse effects of aerosols and toward their health-promoting properties [47,74]. We concur, and predict that the respiratory and gastrointestinal microbiotas of urban dwellers will be found to vary according to the different concentrations of aeronutrients, aeromicrobes, and pollutants in their local environments. It is possible that the availability of aeronutrients influences the growth and survival of aeromicrobes. Research is needed to elucidate the levels and types of aeronutrients and aeromicrobes in differing aerobiomes (natural, indoor, and polluted), the effect of these aerobiomes on the microbiota of the airways and the GIT, and on health and nutrition. It is also not known whether persons with underlying health conditions (e.g. those who are immunocompromised, and suffer from asthma or allergies) respond differently to aeronutrients and aeromicrobes than do healthy individuals.

In conclusion, the concepts of aeronutrients and aeromicrobes offer a transformative perspective on the role of air in human nutrition and health. They challenge conventional wisdoms surrounding nutrient sources and their pathways into the body, with implications for nutrition, public health, environmental science, and even long-duration space travel. Although the concepts of aeronutrients and aeromicrobes are currently supported by multiple lines of evidence, further research is needed to quantify the extent of their contributions to human health. These concepts have the potential improve public health policies so that access to aeronutrients and aeromicrobes can be considered to be an integral part of a healthy lifestyle, and healthcare can incorporate interventions with aeronutrients and aeromicrobes for improving nutrition-related chronic disease.

## Author contributions

Both authors read and approved the final manuscript.

## Conflict of interest

The authors report no conflicts of interest.

## Funding

The authors reported no funding received for this study.
